# Draft genome of the *Arabidopsis thaliana* phyllosphere bacterium, *Williamsia* sp. ARP1

**DOI:** 10.1186/s40793-015-0122-x

**Published:** 2016-01-16

**Authors:** Hannes Horn, Alexander Keller, Ulrich Hildebrandt, Peter Kämpfer, Markus Riederer, Ute Hentschel

**Affiliations:** Department of Botany II, Julius-von-Sachs Institute for Biological Sciences, University of Würzburg, Julius-von-Sachs-Platz 3, D-97082 Würzburg, Germany; GEOMAR Helmholtz Centre for Ocean Research, RD3 Marine Microbiology and Christian-Albrechts University of Kiel, Düsternbrooker Weg 20, D-24105 Kiel, Germany; Department of Animal Ecology and Tropical Biology, Biocenter, University of Würzburg, Am Hubland, D-97074 Germany; Institut für Angewandte Mikrobiologie, Justus-Liebig-Universität Giessen, Heinrich-Buff-Ring 26, D-35392 Giessen, Germany

**Keywords:** Draft genome, Phyllosphere, *Williamsia* sp. ARP1, Adaption, Whole genome sequencing, Next generation sequencing, Assembly, Annotation, *Arabidopsis thaliana*

## Abstract

The Gram-positive actinomycete *Williamsia* sp. ARP1 was originally isolated from the *Arabidopsis thaliana* phyllosphere. Here we describe the general physiological features of this microorganism together with the draft genome sequence and annotation. The 4,745,080 bp long genome contains 4434 protein-coding genes and 70 RNA genes. To our knowledge, this is only the second reported genome from the genus *Williamsia* and the first sequenced strain from the phyllosphere. The presented genomic information is interpreted in the context of an adaptation to the phyllosphere habitat.

**Electronic supplementary material:**

The online version of this article (doi:10.1186/s40793-015-0122-x) contains supplementary material, which is available to authorized users.

## Introduction

The genus *Williamsia* was originally proposed by Kämpfer et al. in 1999 [1] to accommodate an unusual mycolic-acid containing actinomycete. Members of the genus *Williamsia* are Gram-positive, non-spore forming, and form round, orange colonies. Their cell shape is coccoid- or rod-like [2]. The genus *Williamsia* forms a distinct group within actinomycetes of the suborder *Corynebacterineae* [3], which also comprises the genera *Corynebacterium*, *Dietzia*, *Gordonia*, *Mycobacterium*, *Nocardia*, *Rhodococcus*, *Skermania*, *Tsukamurella**and**Turicella*. Based on the mycolic-acid profile with carbon chain lengths ranging from 50 to 56, the genus *Williamsia* is likely to be placed between the genera *Gordonia* and *Rhodococcus* [1]. At the time of writing, only one other draft genome of *Williamsia* sp. D3 was publicly available [4] and nine species of this taxon were recognized with valid scientific names: *Williamsia deligens* [5], *Williamsia faeni* [6], *Williamsia limnetica* [7], *Williamsia marianensis* [8], *Williamsia maris* [9], *Williamsia muralis* [1], *Williamsia phyllosphaerae* [10], *Williamsia serinedens* [11] and *Williamsia sterculiae* [12]. Further this genus has been linked with the degradation of hexahydro-1,3,5-trinitro-1,3,5-triazine in soils as a sole nitrogen source [13], the degradation of carbonyl sulfide in soils [14] and polychlorinated biphenyls in tree habitats [15]. *Williamsia* was isolated from various sources, including indoor building material [1], human blood [5] and following pulmonary infections [16], oil-contaminated and Antarctic soils [4, 11], extreme environments as glacier ice [17], deep sea sediments of the Mariana Trench [8], hay meadows [6], and the rare soil biosphere [18]. Besides, *Williamsia* was also reported as an endophyte of grey box eucalyptus tree roots [19] and as an epiphytic bacterium residing in the phyllosphere of white clover [20].

The phyllosphere, known as the aerial surface of plant leaves, is a short-lived environment [21] to diverse microorganisms of various taxonomic groups comprising bacteria, filamentous fungi, yeasts, viruses and protists. The phyllosphere presents a challenging environment for microbial colonizers with respect to climatic conditions, UV radiation, desiccation, water availability, reactive oxygen species, and in terms of antimicrobial compounds produced by the plant or possibly also microbes [21–25]. Additionally, the wax composition of the cuticle, surface characteristics such as stomata and veins affect nutrient availability and leaching, as they are likely to retain more water [23, 26].

Here, we present a summary, classification and general physiological features of the strain *Williamsia* sp. ARP1 together with the genomic sequencing, assembly, annotation, and its putative adaptions to the phyllosphere.

## Organism information

### Classification and features

**Fig. 1:**
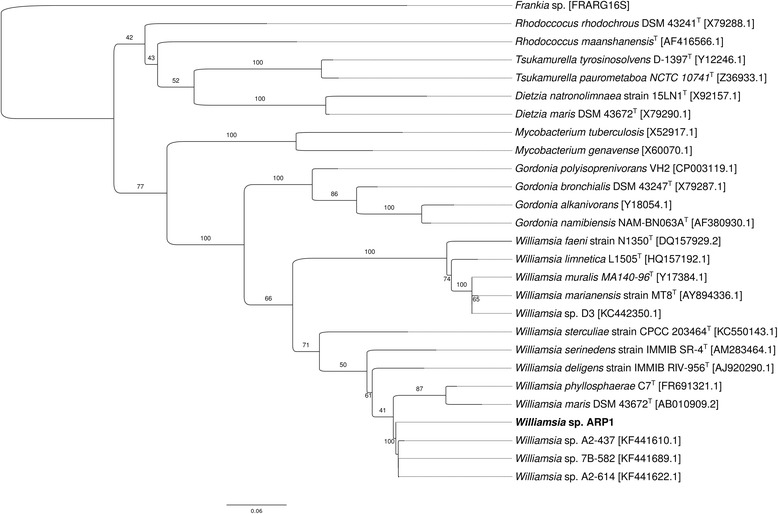
16S rRNA gene based maximum likelihood phylogenetic tree highlighting the position of *Williamsia* sp. ARP1 within the suborder *Corynebacterineae*. The tree is based on 16 s rRNA sequences comprising the genera *Williamsia*, *Gordonia*, *Mycobacterium*, *Dietzia*, *Tsukamurella*, *Rhodococcus* and *Frankia* as an outgroup. The *Williamsia* sp. ARP1 is highlighted in *bold text* to show its position. The maximum-likelihood phylogenetic tree was generated using PhyML with the GTR substitution model. Numbers at the nodes are percentages of 1000 bootstrap replicates. Genbank accession numbers are indicated in *parentheses*; type strains are tagged with a *superscripted T*. The *scale bar* represents 0.06 substitutions per nucleotide position

The genus *Williamsia* belongs to the suborder *Corynebacterineae* [3] of actinomycetes owing to the presence of mycolic acid in the cell wall [2]. Since 2009, it was assigned to the family *Nocardiaceae* [27, 28]. *Williamsia* and other genera of this family form a distinct clade in a 16S rRNA phylogenetic tree as well as by using a combination of phenotypic markers [29]. In order to resolve the taxonomic position of *Williamsia* sp. ARP1, a 16S rRNA sequence (length of 1504 bp) derived from the assembled genome was compared with the NCBI non-redundant and 16S microbial database using BLASTn [30]. The five nearest sequences with the highest identity (all <100 %), the nine validly described *Williamsia* species, as well as representative sequences of the suborder *Corynebacterineae* – *Gordonia*, *Rhodococcus*, *Dietzia*, *Mycobacterium*, *Tsukamurella* and *Turicella* - were used for phylogenetic analysis. A strain of the family *Frankineae* was chosen as the outgroup. All 16S rRNA sequences were aligned using the SINA web aligner (variability profile: Bacteria) [31] and the phylogenetic tree was assessed using PhyML [32] with a generalised time reversible (GTR) substitution model, gamma distribution and 1000 bootstrap replications. All genera formed distinct clades (except *Rhodococcus*) and were well supported by bootstrap values ≥50 %. *Williamsia* formed two well supported distinct clades consisting of five and nine sequences, respectively. Within these clades, however, bootstrap values were weaker, due to low variation between 16S sequences. Closest sequences to *Williamsia* sp. ARP1 were *Williamsia* sp. 7B-582, A2-614 and A2-437 (all three originating from sediment), and phylogeny in this subclade could not be resolved better due to a multifurcation (Fig. 1). All three 16S rRNA gene sequences showed a sequence identity of 99.93 % for strain 7B-582, 99.93 % for strain A2-614, 99.64 % for strain A2-437 to *Williamsia* ARP1. Minimum information about the genome sequence of *Williamsia* sp. ARP1 (MIGS) is provided in Table 1.

**Table 1:** Classification and general features of *Williamsia* sp. ARP1 [34]

MIGS ID	Property	Term	Evidence code^a^
	Classification	Domain *Bacteria*	TAS [73]
		Phylum *Actinobacteria*	TAS [74]
		Class *Actinobacteria*	TAS [3]
		Order *Actinomycetales*	TAS [3, 28, 75, 76]
		Family *Nocardiaceae*	TAS [3, 28, 75, 76]
		Genus *Williamsia*	TAS [1]
		Species *Williamsia* sp.	IDA
		(Type) strain: ARP1	IDA
	Gram stain	Positive	IDA
	Cell shape	Coccoid to rod-like	IDA
	Motility	Non-motile	IDA
	Sporulation	Non-sporulating	IDA
	Temperature range	4–36 °C	IDA
	Optimum temperature	25–30 °C	IDA
	pH range; Optimum	Not reported	NAS
	Carbon source	organic carbon	IDA
MIGS-6	Habitat	Phyllosphere	IDA
MIGS-6.3	Salinity	1.0–6.0 %	IDA
MIGS-22	Oxygen requirement	Aerobic	IDA
MIGS-15	Biotic relationship	Commensal	IDA
MIGS-14	Pathogenicity	Non-pathogenic	NAS
MIGS-4	Geographic location	Würzburg, Germany	IDA
MIGS-5	Sample collection	2012	IDA
MIGS-4.1	Latitude	49.766556	IDA
MIGS-4.2	Longitude	9.931768	IDA
MIGS-4.3	Depth	Plant surface	IDA
MIGS-4.4	Altitude	198 m above sea level	IDA

^a^Evidence codes - *IDA* Inferred from Direct Assay, *TAS* Traceable Author Statement (i.e., a direct report exists in the literature), *NAS* Non-traceable Author Statement (i.e., not directly observed for the living, isolated sample, but based on a generally accepted property for the species, or anecdotal evidence). These evidence codes are from the Gene Ontology project [77]

**Fig. 2:**
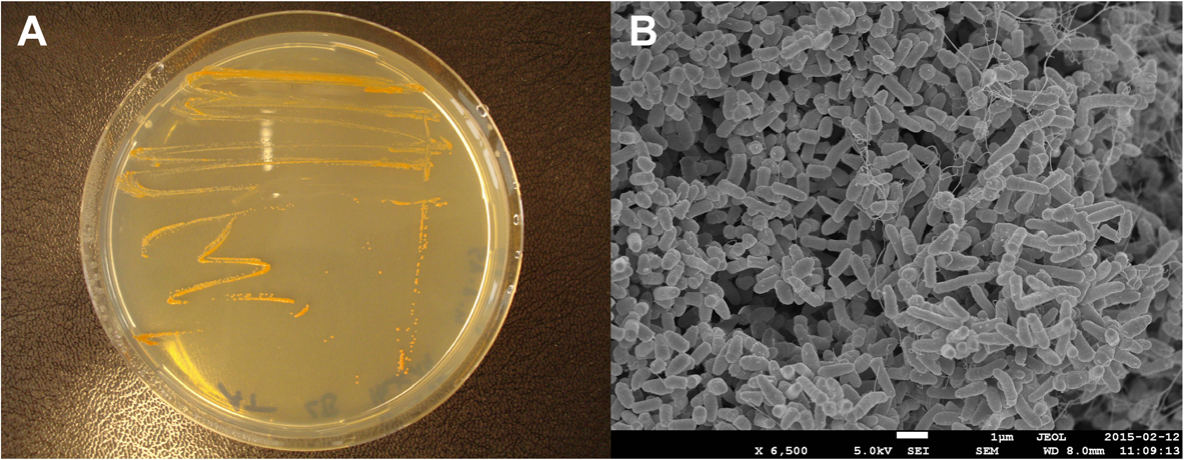
General characteristics of *Williamsia* sp. ARP1. **a** The morphology of the colonies after three days of growth on LB-agar at 30 °C. **b** Image of *Williamsia* sp. ARP1 using scanning electron microscopy

The colonies of *Williamsia* sp. ARP1 were orange to red in color on LB agar medium (Fig. 2a). Strain ARP1 was shown to be Gram-positive by Gram staining (data not shown). The cells of the strain were coccoid to rod-like with a diameter of about 1.0–1.5 μm (Fig. 2b). Further, the strain showed positive oxidase and catalase reaction and an aerobic respiratory metabolism. Cells were growing at a temperature range between 4 and 36 °C. Optimal growth was observed between 25 and 30 °C after 3 days on tryptic soy agar, Reasoner’s 2A agar, and nutrient agar (all Oxoid). NaCl tolerance was investigated at different concentrations of NaCl (0.5–8.0 (*w*/*v*) %) in tryptic soy broth (TSB, Oxoid) with the cells growing in the presence of 1.0–6.0 % NaCl. The strain lacked motility after 3 days of growth in TSB at 30 °C, as observed under the light microscope. In agreement with this observation, a flagellum was not observed which is further backed up by the lack of flagellar genes (i.e., fliX, flgX and motX genes) on its genome. These findings were consistent with previous descriptions for this genus.

## Genome sequencing information

### Genome project history

The organism was selected for sequencing as part of ongoing *Arabidopsis* phyllosphere microbiology studies [33]. The sequencing project was completed in July 2014 and sequencing data was deposited as a Whole Genome Shotgun (WGS) project in Genbank under the BioProject PRJNA272726 and the accession number JXYP00000000 consisting of 50 contigs (≥1000 bp). The genome sequencing was carried out with a MiSeq (Illumina Inc.) located in-house at our University. A summary of the project information according to the MIGS version 2.0 is shown in Table 2 [34].

**Table 2:** Project information

MIGS ID	Property	Term
MIGS 31	Finishing quality	Draft genome
MIGS-28	Libraries used	One Illumina paired-end library (400 bp insert size)
MIGS 29	Sequencing platforms	Illumina MiSeq
MIGS 31.2	Fold coverage	65×
MIGS 30	Assemblers	SPAdes 3.0, SSPACE 3.0
MIGS 32	Gene calling method	Prodigal 2.6.1
	Genbank ID	JXYP00000000
	Locus Tag	TU34
	GenBank Date of Release	July 1, 2015
	GOLD ID	Gp0118481
	BIOPROJECT	PRJNA272726
MIGS 13	Source Material Identifier	DSM 46827
	Project relevance	Phyllosphere, Environmental

### Growth conditions and genomic DNA preparation

Several plants were collected from a Landsberg *erecta* (L*er*) population of *Arabidopsis thaliana* from the Botanical Garden (University of Würzburg, June 2012). Leaf washings [35] were used for inoculation of minimal media with C_16_ alkane (Sigma-Aldrich) as the sole carbon source in order to enrich for bacteria with the ability to degrade long-chain hydrocarbons. Aliquots were streaked (in duplicate) on agar plates prepared with minimal media and supplemented with C_22_ alkane (Sigma-Aldrich). This procedure provided a total of 17 isolates, of which most belonged to the genus *Rhodococcus* and two to genus *Williamsia* [33].

*Williamsia* sp. ARP1 was grown in 10 ml Luria-Bertani broth medium (10 g peptone, 5 g yeast extract, 5 g NaCl in 1000 ml demineralized water) for 24 h at 30 °C and rotary shaking at 180 rpm. For genomic DNA isolation, 2 ml of overnight culture were centrifuged at 8000 rpm for 5 min at room temperature. The pellet was rinsed in 1 ml TNE (1 ml 1 M Tris pH 8, 0.2 ml 5 M NaCl, 2 ml 0.5 M EDTA pH8, and 100 ml demineralized water) and resuspended in 270 μl TNEx (TNE, 1 % *v*/*v* TritonX-100) and 25 μl lysozyme (10 mg/ml). After a 30 min incubation at 37 °C, 50 μl of proteinase K (20 mg/ml) were added. After an incubation of 2 h and 55 °C, 15 μl of 5 M NaCl and 500 μl of 100 % EtOH were added. The mixture was then centrifuged at 13,000 rpm for 15 min at room temperature, rinsed with 70 % EtOH, air dried and resuspended in 150 μl TE buffer. The quality and quantity of the extracted DNA was evaluated by 0.8 % (*w*/*v*) agarose gel electrophoresis, by measuring absorption ratios 260/280 and 260/230 with a Nanodrop 2000c Spectrophotometer (Thermo Fisher Scientific) and an additional Qubit dsDNA HS assay (Life Technologies).

### Genome sequencing and assembly

High molecular weight DNA was cleaned with the DNA Clean & Concentrator kit (Zymo Research). The genomic DNA library for the Illumina platform was generated using Nextera XT (Illumina Inc.) according to the manufacturer’s instructions. After tagmentation, size-selection was performed using NucleoMag NGS Clean-up and Size Select (Macherey-Nagel) to obtain a library with median insert-size around 400 bp. After PCR enrichment, the library was validated with a high-sensitivity DNA chip and Bioanalyzer 2100 (both Agilent Technologies, Inc.) and additionally quantified using the Qubit dsDNA HS assay (Life Technologies). Sequencing was performed on a MiSeq device using v2 2 × 250 bp chemistry, and the genome was multiplexed together with ten other bacterial genomes from other sources. Multiplexing was done via dual indexing, with the official Nextera indices N706 and S503 for *Williamsia* sp. ARP1.

In total, 1,304,294 (mean length 237.86 bp) raw paired-end sequences were subjected to the Trimmomatic software [36] for adapter and quality trimming (mean Phred quality score ≥30), filtering of sequences containing ambiguous bases and a minimum length of 200 bp. Subsequently, human and viral decontamination was excluded using DeconSeq [37]. The 1,287,247 (mean length 236.95 bp) remaining paired-end sequences were assembled with five different tools: a5-miseq [38], IDBA-UD [39], MaSuRCA [40], SPAdes [41] and Velvet [42]. In order to obtain the most reliable contigs, all assemblies were evaluated with QUAST [43], REAPR [44], ALE [45] and Feature Response Curves [46]. According to those evaluations, we have selected SPAdes assembler with enabled pre-correction and k-mer sizes ranging from 15 to 125 (step size of 10) as the best assembly. Obtained contigs were extended with remaining reads where possible. This led to 50 large contigs (≥1000 bp, N_50_: 140,970 bp, longest contig: 428,355 bp) and an overall genome size of 4,745,080 bp (GC content: 68.63 %). As a final step, the contigs were ordered according to the nearest related complete genome by functional content using Mauve in 12 iterations [47]. As *Williamsia* sp. D3 was only available as a draft genome, *Gordonia bronchialis* was used for this step.

### Genome annotation

Open reading frames were identified using Prodigal [48] followed by manual correction. The predicted coding sequences were translated into amino acid sequences and searched against COG position-specific scoring matrices obtained from the Conserved Domains Database [49] using RPS-BLAST [30]. Comparisons with TIGRFAM, Pfam, and PANTHER databases were performed with the InterProScan pipeline [50]. Only matches with an e-value ≤1∗10^−2^, ≥25 % identity and a minimum of 70 % alignment length to the target sequence were maintained. During this run, matches were also mapped to Gene Ontology terms. Additional gene prediction and functional annotation was performed with the Integrated Microbial-Genomes Expert Review [51] and the Rapid Annotation using Subsystem Technology webserver [52, 53]. Features as tRNA, rRNA, ncRNA, transmembrane helices, signal peptides, CRISPR elements and secondary metabolite gene clusters were predicted using tRNAscan-SE [54], RNAmmer [55], INFERNAL [56] and Prokka’s prokaryotic RNA covariance models [57], TMHMM [58], SignalP [59] PILER-CR [60] and antiSMASH [61]. Searching for essential genes [62] was performed using HMMER3 [63]. Ortholog detection between *Williamsia* sp. ARP1 and three other genomes were carried out with InParanoid [64] whereas the mean percentage of nucleotide identity among the found orthologous genes was calculated using BLASTn. Average nucleotide identities between *Williamsia* sp. ARP1 and reference genomes were calculated with JSpecies [65].

## Genome properties

**Fig. 3:**
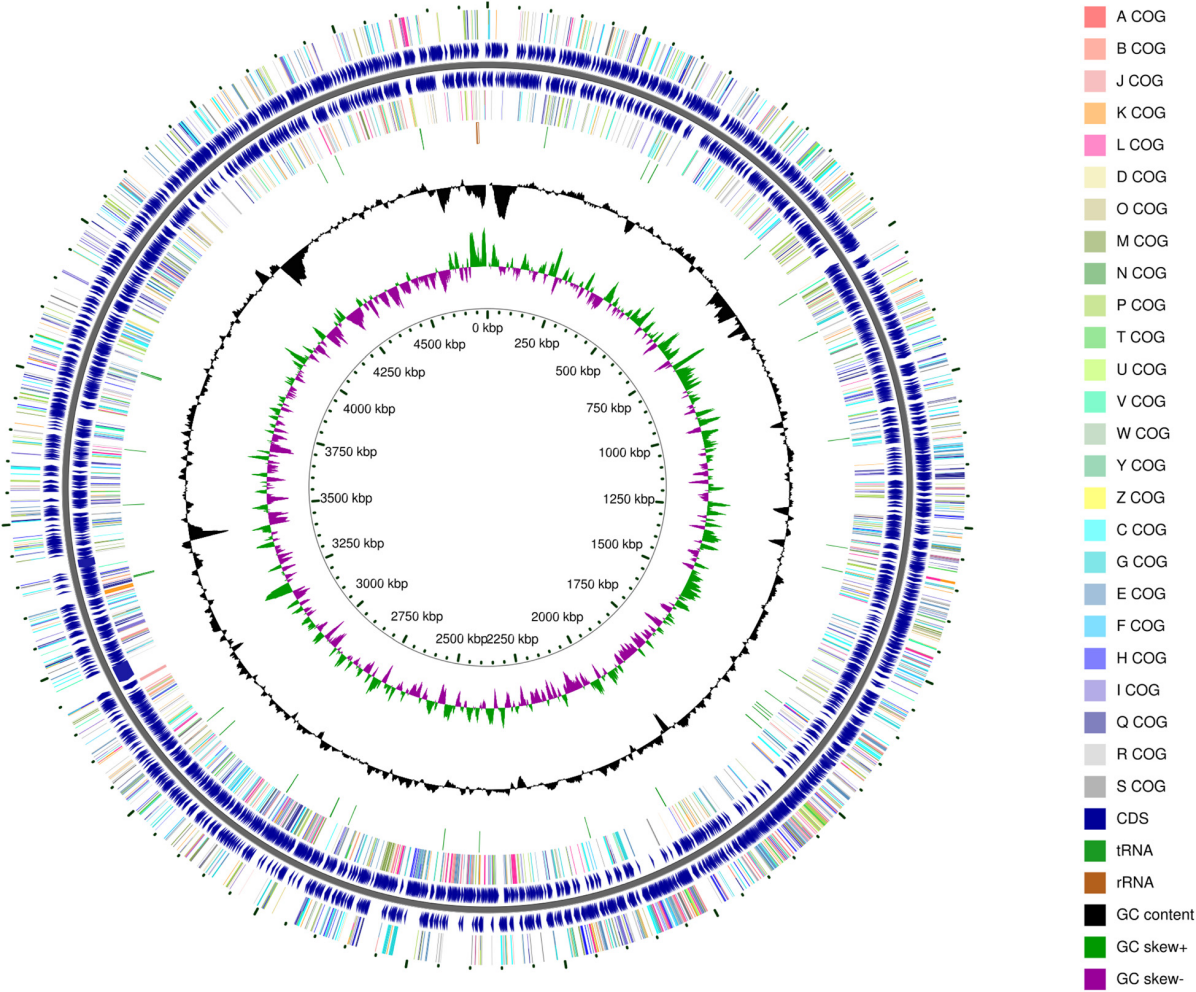
Graphical circular map of the *Williamsia* sp. ARP1 genome. Starting from the outmost circle and moving inwards, each ring of the circle contains information of the genome: genes on the forward strand (*colored* according to their COG categories), CDS on the forward strand (*blue arrows*), CDS on the reverse strand (*blue arrows*), genes on the reverse strand (*colored* according to their COG categories), tRNA and rRNA genes on both strands (*green* and *orange*), GC content (*black*), GC skew (*green* and *purple*) and genome region by kbp

**Fig. 4:**
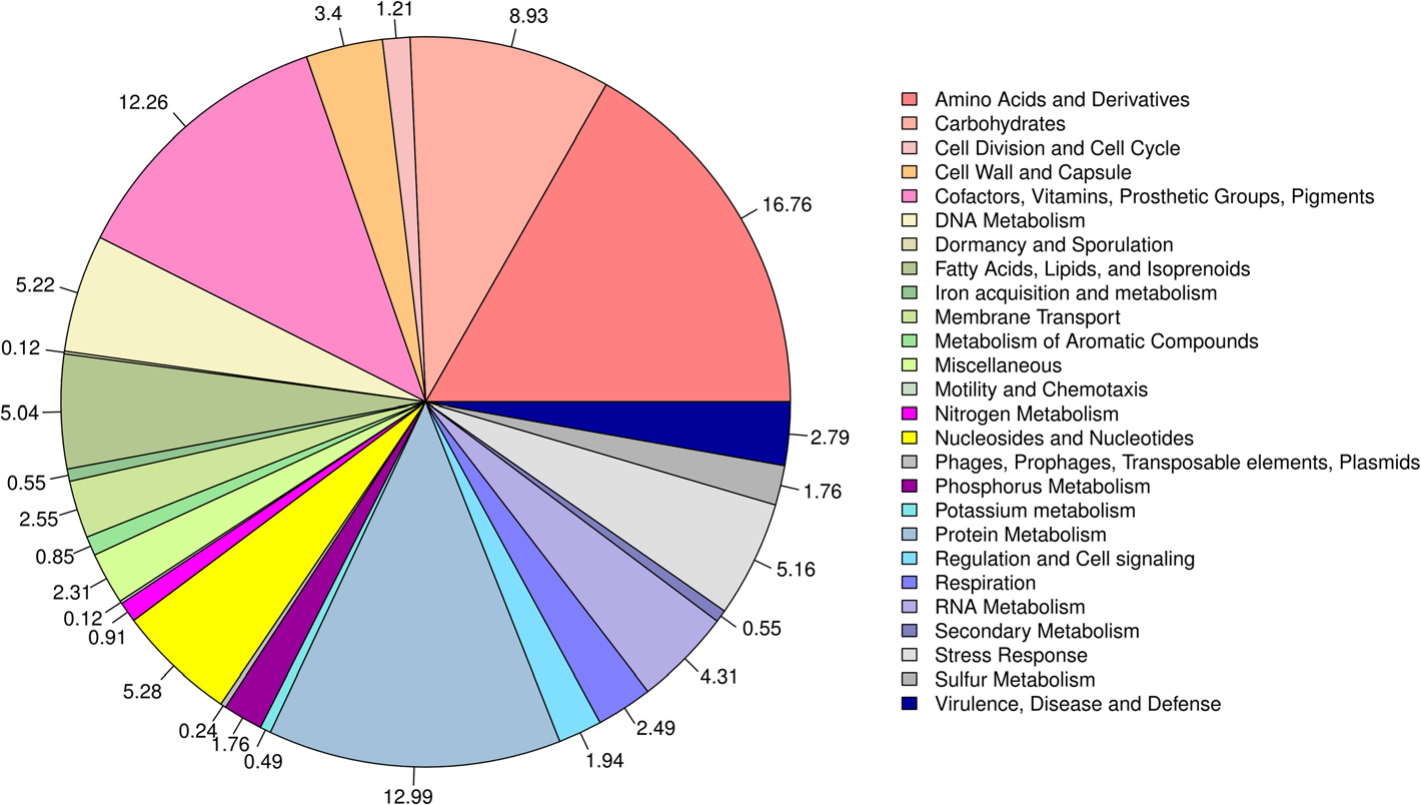
Metabolic subsystems of *Williamsia* sp. ARP1 annotated through the RAST webserver

The *Williamsia* sp. ARP1 draft genome sequence contained a total of 4,745,080 bp distributed over 50 large contigs (≥1000 bp) with an average GC content of 68.63 %. Of the 4509 predicted genes, 4438 (98.42 %) were protein-coding, and 3505 (77.73 %) annotated with putative function. Pseudogenes were not detected. Genes not linked to a function were annotated as hypothetical or unknown function. Of these, 45 belonged to tRNA genes, 21 to ncRNA genes and five to rRNA genes (Table 3). One operon comprising a 16S rRNA, a 5S rRNA and a 23S rRNA gene was found. However two additional 5S rRNA genes suggest the presence of at least three rRNA operons. Functional assignments using COGs, a total of 2204 (59.59 %) of the coding sequences were classified into 23 different classes (Table 4, Fig. 3). Using TIGRFAM or Pfam, 793 (17.59 %) and 1330 (29.50 %) of the sequences could be classified (Table 3). For testing the genome completeness, a set of 111 essential gene markers was searched and 106 (=95.50 %) of them were present in *Williamsia* sp. ARP1. Except two marker genes (ribosomal proteins bS18 and bl28), all of them were found only once (Additional file 1). Within the RAST annotation, 1625 sequences were assigned to 402 metabolic subsystems. The highest ranking among the metabolic subsystems are linked to amino acids and derivatives (8.41 %), cofactors, vitamins and pigments (6.25 %), carbohydrates (5.77 %), protein metabolism (5.61 %), fatty acids, and lipids and isoprenoids (4.32 %) followed by stress response (2.86 %), (Fig. 4).

**Table 3:** Genome statistics

Attribute	Value	% of total
Genome size (bp)	4,745,080	100.00
DNA coding (bp)	4,347,123	91.61
DNA G+C (bp)	3,256,678	68.63
DNA scaffolds	50	
Total genes	4509	100.00
Protein coding genes	4438	98.42
RNA genes	71	1.57
tRNA genes	45	1.00
rRNA genes	5	0.01
rRNA operons	1^a^	
Pseudo genes	0	0.00
Genes in internal clusters	NA	
Genes with function prediction	3505	77.73
Genes assigned to COGs	2207	48.95
Genes with Pfam domains	1330	29.50
Genes with TIGRFAM domains	793	17.59
Genes with signal peptides	334	7.41
Genes with transmembrane helices	1140	25.28
CRISPR repeats	2	0.04

^a^Only one RNA operon appears to be complete

## Insights from the genome sequence

**Fig. 5:**
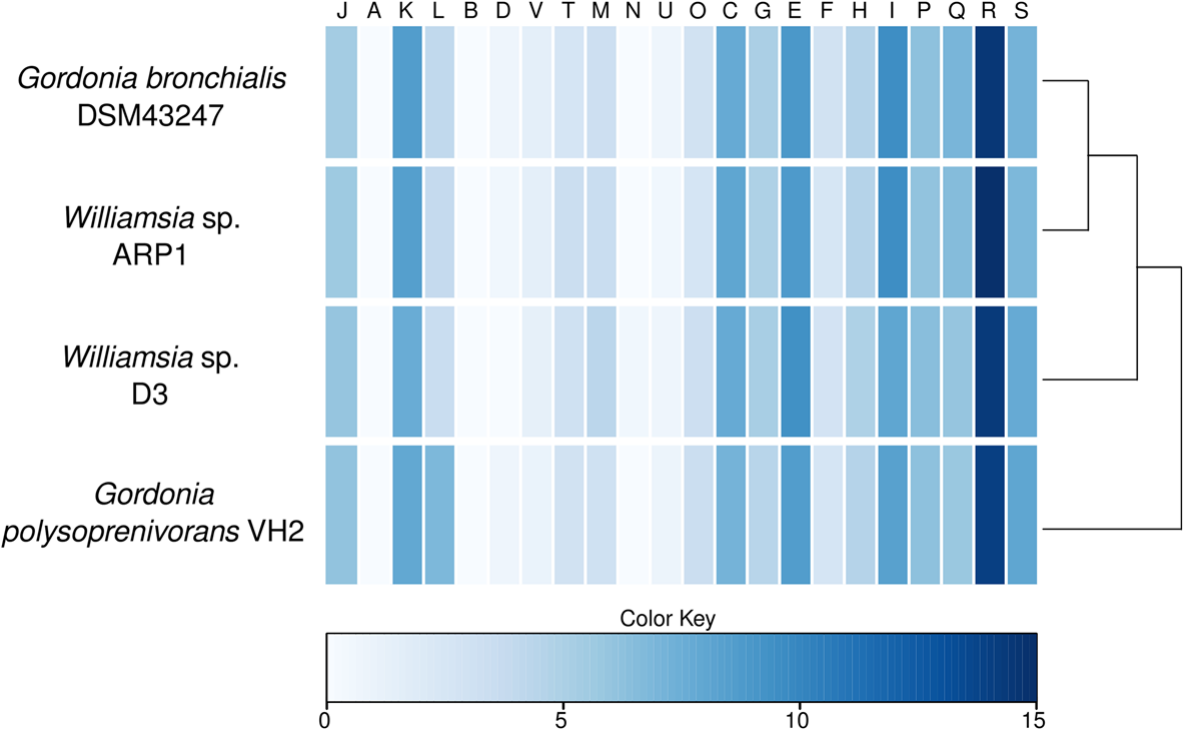
Comparison of COG classes between strain ARP1 and reference genomes. The *color keys* provide the relative percentage of each COG class per genome. The dendrogram is based on correlation analysis

The genome of *Williamsia* sp. ARP1 was smaller but displayed a higher CG content (68.63 %) than its nearest relative genomes (Table 5), thus rendering this genome more similar to the *G. bronchialis* and *G. polysoprenivorans* VH2 (67.00 and 66.96 %) than to *Williamsia* sp. D3 (64.60 %) (Table 5). Considering the similarity between 16S rRNA sequences and its placement in the phylogenetic tree, strain ARP1 was however clearly assigned to the genus *Williamsia* (Fig. 1). With respect to orthologous genes, *Williamsia* sp. D3 was found to be the most similar strain to *Williamsia* sp. ARP1 with an average nucleotide identity of these orthologs of 75.53 %. Notably, the differences between *Williamsia* sp. ARP1 and the *Gordonia* strains and VH2 (75.17 and 74.84 % identity, respectively) is similar to the difference between the two *Williamsia* strains (75.53 %), (Additional file 2). Neither the clustering of COG classes nor the average nucleotide identities (ANI) were discriminative between the two genera (Fig. 5, Additional file 3). The ANI values are noticeably lower than the calculated cut-off values for species level identification (95) [66].

**Table 4:** Number of genes associated with general COG functional categories

Code	Value	% age	Description
J	143	3.17	Translation, ribosomal structure, and biogenesis
A	1	0.02	RNA processing and modification
K	183	4.06	Transcription
L	85	1.89	Replication, recombination, and repair
B	1	0.02	Chromatin structure and dynamics
D	0	0.00	Cell cycle control, Cell division, chromosome partitioning
V	31	0.69	Defense mechanisms
T	74	1.64	Signal transduction mechanisms
M	102	2.26	Cell wall/membrane biogenesis
N	11	0.24	Cell motility
U	18	0.40	Intracellular trafficking and secretion
O	79	1.75	Posttranslational modification, protein turnover, chaperones
C	184	4.08	Energy production and conversion
G	125	2.77	Carbohydrate transport and metabolism
E	226	5.01	Amino acid transport and metabolism
F	66	1.46	Nucleotide transport and metabolism
H	118	2.62	Coenzyme transport and metabolism
I	194	4.30	Lipid transport and metabolism
P	154	3.42	Inorganic ion transport and metabolism
Q	141	3.13	Secondary metabolites biosynthesis, transport and catabolism
R	346	7.67	General function prediction only
S	184	4.08	Function unknown
-	2231	49.48	Not in COGs

The total is based on the total number of protein coding genes in the genome

**Table 5:** Used actinomycete reference genomes in this study

Species	Strain	Accession number	Genome Size [Mbp]	G+C content
*Williamsia* sp.	D3	NZ_AYTE000000000.1	5.62	64.60
*Gordonia bronchialis*		CP001802.1	5.21	67.00
*G. polysoprenivorans*	VH2	NC_016906.1	5.67	66.96

### Extended insights

#### UV radiation

UV radiation may impose stress on bacteria inhabiting plant leaves. In this context, a cluster of genes synthesizing mycosporins was found. These secondary metabolites are known to protect cells by absorbing UV light without generating reactive oxygen species (ROS) [67, 68]. Additionally, genes involved in the repair of UV-damaged DNA were found, which comprise DNA photolyases, the UvrABC endonuclease enzyme complex, and the DNA helicase II UvrD of the UvrABC system. The red color of *Williamsia* sp. ARP1 might protect it against photo-oxidative stress as pigmentation is known to be a common feature of phyllosphere colonizers [69]. All genes of the carotenoid biosynthetic pathway were found, consisting of a geranylgeranyl diphosphate synthase, a phytoene synthase, a phytoene desaturase, a carotene desaturase and a lycopene-β-cyclase. The products of this pathway are lycopene and β-carotene, both producing orange to red pigments.

#### Oxidative stress

Further adaptions to an epiphytic lifestyle are encoded on genes responding to reactive oxygen species (ROS; e.g. hydrogen peroxide, superoxide, hydroperoxil radical), which are products of the plant defense [70, 71]. Here, two genes encoding for glutathione peroxidases, two superoxide dismutases with copper/zinc or manganese as active site, two glutaredoxins, three thioredoxins, and one catalase were found.

#### Temperature shifts

Regarding temperature shifts, the heatshock chaperones DnaK, DnaJ and GrpE and the cold shock protein CspC were identified.

#### Uptake

ABC transporters for the uptake of carbohydrates such as ribose, glycerol or maltose, amino acids such as methionine, known plant photosynthates such as fructose, and enzymes for fructose utilization were identified. Also, genes mediating the uptake of choline and subsequent biosynthesis (choline dehydrogenase, betaine-aldehyde dehydrogenase) of the osmoprotectant betaine were found.

#### Desiccation

Trehalose is a compatible solute and known to prevent cells from desiccation and water loss [72]. Eight genes encoding for the biosynthesis pathway (Malto-oligosyltrehalose synthase, 1,4-alpha-glucan (glycogen) branching enzyme, GH-13-type trehalose-6-phosphate phosphatase, putative glucanase glgE, malto-oligosyltrehalose trehalohydrolase, glycogen debranching enzyme alpha, alpha-trehalose-phosphate synthase, glucoamylase) were identified.

## Conclusions

The isolate ARP1 was isolated from the *Arabidopsis thaliana* phyllosphere. Phylogenetic analysis based on the 16S rRNA gene confirmed its affiliation to the genus *Williamsia*. However genomic properties also showed close similarities to *Gordonia*, as derived from GC content, COGs, and average nucleotide identities. Thus, an unequivocal delinearization based on the functional genomics level was not possible, which may be due to the underrepresentation of genomes from this genus. The genomic features of strain ARP1 would be consistent with a lifestyle within the phyllosphere, including putative adaptions to UV radiation, heat and cold shock, desiccation and oxidative stress. With this study, we provide novel genomic insights into the rarely sequenced genus *Williamsia* and discuss its putative adaptations to the phyllosphere habitat.

## Additional files

Additional file 1:
**Identified essential genes in the** ***Williamsia*** **sp. ARP1 genome.** (PDF 75 kb) Additional file 2:
**Orthologous gene comparison of** ***Williamsia*** **sp. ARP1 and three other actinomycete genomes.** (PDF 58 kb) Additional file 3:
**Average nucleotide identities between** ***Williamsia*** **sp. ARP1 and nearest actinomycete genomes.** (PDF 54 kb)
