# Supplementary material for: Draft genome of the Arabidopsis thaliana phyllosphere bacterium, Williamsia sp. ARP1
Source: Stand Genomic Sci. 2016 Jan 16;11:8. doi: 10.1186/s40793-015-0122-x (PMC4715301; doi:10.1186/s40793-015-0122-x)
Supplement: Additional file 1: — Identified essential genes in the Williamsia sp. ARP1 genome. (PDF 75 kb) [file 40793_2015_122_MOESM1_ESM.pdf]

**Additional Table S1** - Found essential genes in the genome of *Williamsia* sp. ARP1. Open reading frames (ORFs) were mapped to Pfam and TIGRFAM domains using hmmer3. Given in the table is the number of the ORF, the e–value and bitscore of the matched domain and its description.

| Open Reading Frame | e-value | bitscore | TIGRFAM/Pfam | Description |
|---|---|---|---|---|
| contig10_size140970_116 | 0 | 1679.5 | TIGR02386 | DNA-directed RNA polymerase beta' subunit |
| contig10_size140970_117 | 0 | 1699 | TIGR02013 | DNA-directed RNA polymerase beta subunit |
| contig10_size140970_129 | 2.1e-48 | 161.8 | TIGR00855 | ribosomal protein bL12 |
| contig10_size140970_130 | 5.5e-27 | 91.9 | PF00466.15 | Ribosomal protein L10 |
| contig10_size140970_29 | 9.7e-68 | 224.9 | TIGR01029 | ribosomal protein uS7 |
| contig10_size140970_30 | 7e-67 | 220.9 | TIGR00981 | ribosomal protein uS12 |
| contig11_size140457_43 | 2.1e-131 | 436.4 | TIGR00092 | GTP-binding protein YchF |
| contig11_size140457_72 | 2.6e-56 | 187.6 | TIGR00086 | SsrA-binding protein |
| contig12_size136193_35 | 4e-10 | 37.8 | TIGR01031 | ribosomal protein bL32 |
| contig12_size136193_38 | 1.4e-24 | 84.3 | TIGR00009 | ribosomal protein bL28 |
| contig12_size136193_41 | 1.9e-24 | 83.6 | TIGR00165 | ribosomal protein bS18 |
| contig14_size119322_19 | 5e-62 | 206.7 | TIGR03263 | guanylate kinase |
| contig14_size119322_25 | 5.9e-65 | 217.4 | TIGR00460 | methionyl-tRNA formyltransferase |
| contig14_size119322_39 | 5.2e-141 | 467.9 | PF00162.14 | Phosphoglycerate kinase |
| contig14_size119322_41 | 3.7e-20 | 69.7 | TIGR00810 | preprotein translocase SecG subunit |
| contig18_size111977_99 | 8.4e-125 | 415 | TIGR00414 | serine–tRNA ligase |
| contig1_size428335_118 | 4.5e-242 | 803.8 | TIGR00392 | isoleucine–tRNA ligase |
| contig1_size428335_13 | 5.1e-171 | 568.6 | TIGR00472 | phenylalanine–tRNA ligase beta subunit |
| contig1_size428335_136 | 1.6e-98 | 328 | PF01795.14 | MraW methylase family |
| contig1_size428335_1 | 3e-107 | 357.1 | TIGR00234 | tyrosine–tRNA ligase |
| contig1_size428335_14 | 4.5e-85 | 283.7 | TIGR00468 | phenylalanine–tRNA ligase alpha subunit |
| contig1_size428335_16 | 8.2e-42 | 140.1 | TIGR01032 | ribosomal protein bL20 |
| contig1_size428335_17 | 3.7e-26 | 89.2 | TIGR00001 | ribosomal protein bL35 |
| contig1_size428335_18 | 3.3e-60 | 200.5 | TIGR00168 | translation initiation factor IF-3 |
| contig1_size428335_25 | 0 | 1073.5 | TIGR00631 | excinuclease ABC subunit B |
| contig1_size428335_264 | 2.4e-112 | 373.8 | TIGR01391 | DNA primase |
| contig1_size428335_268 | 9.2e-150 | 497.8 | TIGR00389 | glycine–tRNA ligase |
| contig1_size428335_27 | 1.9e-46 | 156.2 | TIGR00152 | dephospho-CoA kinase |

**Additional Table S1** - Continued

| Open Reading Frame | e-value | bitscore | TIGRFAM/Pfam | Description |
|---|---|---|---|---|
| contig1_size428335_275 | 4.7e-67 | 224.1 | TIGR00436 | GTP-binding protein Era |
| contig1_size428335_277 | 2.5e-33 | 112.5 | TIGR00043 | rRNA maturation RNase YbeY |
| contig1_size428335_300 | 4e-292 | 967.8 | TIGR01393 | elongation factor 4 |
| contig1_size428335_326 | 2.5e-24 | 83.6 | TIGR00029 | ribosomal protein bS20 |
| contig1_size428335_341 | 3.9e-23 | 79.7 | TIGR00009 | ribosomal protein bL28 |
| contig21_size94691_14 | 2.9e-263 | 873.8 | TIGR00396 | leucine–tRNA ligase |
| contig21_size94691_77 | 9.9e-17 | 58.9 | TIGR01030 | ribosomal protein bL34 |
| contig21_size94691_78 | 3.7e-147 | 488.8 | TIGR00362 | chromosomal replication initiator protein DnaA |
| contig21_size94691_79 | 7.7e-103 | 342.2 | TIGR00663 | DNA polymerase III beta subunit |
| contig21_size94691_83 | 1.4e-286 | 950.1 | TIGR01059 | DNA gyrase B subunit |
| contig21_size94691_84 | 0 | 1164.1 | TIGR01063 | DNA gyrase A subunit |
| contig22_size93302_21 | 2.3e-235 | 780.9 | TIGR00575 | DNA ligase NAD-dependent |
| contig22_size93302_25 | 1.8e-84 | 281.6 | TIGR00420 | tRNA (5-methylaminomethyl-2-thiouridylate)-methyltransferase |
| contig24_size85376_38 | 6.6e-129 | 428.9 | TIGR00435 | cysteine–tRNA ligase |
| contig25_size81038_1 | 4.6e-117 | 389.3 | TIGR00115 | trigger factor |
| contig25_size81038_15 | 2.1e-26 | 90.3 | TIGR00061 | ribosomal protein bL21 |
| contig25_size81038_16 | 2e-35 | 118.7 | TIGR00062 | ribosomal protein bL27 |
| contig25_size81038_17 | 3.4e-120 | 398.9 | TIGR02729 | Obg family GTPase CgtA |
| contig25_size81038_30 | 6.5e-65 | 216.7 | TIGR02191 | ribonuclease III |
| contig25_size81038_42 | 2e-102 | 340.2 | TIGR00064 | signal recognition particle-docking protein FtsY |
| contig25_size81038_46 | 4.9e-176 | 583.7 | TIGR00959 | signal recognition particle protein |
| contig25_size81038_48 | 1.5e-29 | 99.8 | TIGR00002 | ribosomal protein bS16 |
| contig25_size81038_53 | 2.7e-50 | 167.2 | TIGR01024 | ribosomal protein bL19 |
| contig25_size81038_70 | 2.9e-108 | 358.3 | TIGR01011 | ribosomal protein uS2 |
| contig25_size81038_71 | 2.3e-75 | 251.5 | TIGR00116 | translation elongation factor Ts |
| contig25_size81038_73 | 1.2e-64 | 215.1 | TIGR00496 | ribosome recycling factor |
| contig25_size81038_9 | 4.6e-287 | 952.5 | TIGR00422 | valine–tRNA ligase |
| contig26_size76862_38 | 8.4e-39 | 131.3 | TIGR00158 | ribosomal protein bL9 |
| contig26_size76862_39 | 1.4e-25 | 87.2 | TIGR00165 | ribosomal protein bS18 |
| contig26_size76862_41 | 1e-26 | 90.9 | TIGR00166 | ribosomal protein bS6 |

**Additional Table S1** - Continued

| Open Reading Frame | e-value | bitscore | TIGRFAM/Pfam | Description |
|---|---|---|---|---|
| contig28_size60006_10 | 5.8e-62 | 206.8 | PF00573.17 | Ribosomal protein L4/L1 |
| contig28_size60006_11 | 6.4e-45 | 151.8 | PF00297.17 | Ribosomal protein L3 |
| contig28_size60006_12 | 4.3e-46 | 153.2 | TIGR01049 | ribosomal protein uS10 |
| contig28_size60006_2 | 3.6e-29 | 98.9 | PF00366.15 | Ribosomal_S17 |
| contig28_size60006_3 | 4.3e-20 | 69.5 | TIGR00012 | ribosomal protein uL29 |
| contig28_size60006_4 | 2.6e-56 | 187.1 | TIGR01164 | ribosomal protein uL16 |
| contig28_size60006_5 | 7.5e-94 | 311.3 | TIGR01009 | ribosomal protein uS3 |
| contig28_size60006_6 | 3.1e-48 | 160.3 | TIGR01044 | ribosomal protein uL22 |
| contig28_size60006_7 | 2.2e-47 | 157.3 | TIGR01050 | ribosomal protein uS19 |
| contig28_size60006_8 | 4.4e-127 | 421.1 | TIGR01171 | ribosomal protein uL2 |
| contig28_size60006_9 | 1.1e-27 | 94 | PF00276.15 | Ribosomal protein L23 |
| contig29_size50806_15 | 8.3e-43 | 143.2 | TIGR00059 | ribosomal protein bL17 |
| contig29_size50806_16 | 3.7e-114 | 378.8 | TIGR02027 | DNA-directed RNA polymerase alpha subunit |
| contig29_size50806_17 | 1.2e-79 | 264.6 | TIGR01017 | ribosomal protein uS4 |
| contig29_size50806_18 | 4.9e-49 | 163.4 | PF00411.14 | ribosomal protein L11 |
| contig29_size50806_19 | 4.1e-37 | 125.1 | PF00416.17 | ribosomal protein S13 |
| contig29_size50806_28 | 3.9e-130 | 432.4 | TIGR00967 | preprotein translocase SecY subunit |
| contig29_size50806_29 | 8.1e-46 | 153.4 | TIGR01071 | ribosomal protein uL15 |
| contig29_size50806_31 | 2.8e-68 | 226.6 | TIGR01021 | ribosomal protein uS5 |
| contig29_size50806_32 | 4.9e-38 | 128 | TIGR00060 | ribosomal protein uL18 |
| contig29_size50806_33 | 5.8e-44 | 146.7 | PF00347.18 | Ribosomal protein L6 |
| contig29_size50806_34 | 2.8e-47 | 158 | PF00410.14 | Ribosomal_S8 |
| contig29_size50806_39 | 2.2e-23 | 80.1 | PF00281.14 | Ribosomal_L5 |
| contig29_size50806_40 | 1.7e-34 | 116.3 | TIGR01079 | ribosomal protein uL24 |
| contig29_size50806_41 | 1.8e-57 | 190.5 | TIGR01067 | ribosomal protein uL14 |
| contig2_size397101_171 | 2.6e-58 | 193.8 | TIGR01066 | ribosomal protein uL13 |
| contig2_size397101_172 | 1e-44 | 149.9 | PF00380.14 | Ribosomal_S9 |
| contig2_size397101_360 | 3.7e-104 | 346.6 | PF00750.14 | tRNA synthetases class I |
| contig2_size397101_368 | 1.9e-144 | 479 | TIGR00019 | peptide chain release factor 1 |
| contig35_size31300_23 | 4.4e-167 | 555.3 | TIGR00409 | proline–tRNA ligase |

**Additional Table S1** - Continued

| **Open Reading Frame** | **e-value** | **bitscore** | **TIGRFAM/Pfam** | **Description** |
|---|---|---|---|---|
| contig35_size31300_27 | 5.9e-106 | 352.2 | TIGR01953 | transcription termination factor NusA |
| contig4_size250961_209 | 2.3e-154 | 512.5 | TIGR03594 | ribosome-associated GTPase EngA |
| contig4_size250961_209 | 3.9e-41 | 139.1 | TIGR00436 | GTP-binding protein Era |
| contig4_size250961_217 | 2.7e-206 | 684.1 | TIGR00337 | CTP synthase |
| contig6_size212888_160 | 2.4e-32 | 110 | PF01025.14 | GrpE |
| contig6_size212888_161 | 3.7e-276 | 915.1 | TIGR02350 | chaperone protein DnaK |
| contig6_size212888_62 | 1.2e-30 | 104.8 | TIGR02432 | tRNA(Ile)-lysidine synthetase |
| contig7_size201016_10 | 5.4e-40 | 133.6 | TIGR00952 | ribosomal protein uS15 |
| contig7_size201016_1 | 1.3e-222 | 738.7 | TIGR00487 | translation initiation factor IF-2 |
| contig7_size201016_150 | 1.1e-188 | 626.6 | TIGR00418 | threonine–tRNA ligase |
| contig7_size201016_172 | 2.2e-131 | 436.5 | TIGR00442 | histidine–tRNA ligase |
| contig7_size201016_175 | 2.2e-209 | 694.6 | TIGR00459 | aspartate–tRNA ligase |
| contig7_size201016_182 | 1.2e-241 | 802.4 | TIGR00344 | alanine–tRNA ligase |
| contig7_size201016_2 | 4.8e-20 | 70.2 | TIGR00082 | ribosome-binding factor A |
| contig7_size201016_49 | 2.9e-172 | 570.2 | TIGR02012 | protein RecA |
| contig8_size193540_171 | 4.9e-111 | 369.3 | TIGR02397 | DNA polymerase III subunit gamma and tau |
| contig9_size150680_4 | 7.6e-99 | 327.6 | TIGR01169 | ribosomal protein uL1 |
| contig9_size150680_5 | 1.6e-62 | 207.5 | TIGR01632 | ribosomal protein uL11 |
| contig9_size150680_6 | 7e-64 | 212.7 | TIGR00922 | transcription termination/antitermination factor NusG |
| contig9_size150680_7 | 3.2e-19 | 66.6 | TIGR00964 | preprotein translocase SecE subunit |
