# Supplementary material for: Draft genome of the Arabidopsis thaliana phyllosphere bacterium, Williamsia sp. ARP1
Source: Stand Genomic Sci. 2016 Jan 16;11:8. doi: 10.1186/s40793-015-0122-x (PMC4715301; doi:10.1186/s40793-015-0122-x)
Supplement: Additional file 2: — Orthologous gene comparison of Williamsia sp. ARP1 and three other actinomycete genomes. (PDF 58 kb) [file 40793_2015_122_MOESM2_ESM.pdf]

Additional Table S2 - Orthologous gene comparison of *Williamsia* sp. ARP1 and three other actinomycete genomes.

**Species**	***Williamsia* sp. ARP1**	***Williamsia* sp. D3**	***Gordonia bronchialis* DSM 43234**	***Gordonia polyisoprenivorans* VH2**
***Williamsia* sp. ARP1**	**4,438**	2,768	2,726	2,818
***Williamsia* sp. D3**	75.53	**4,930**	2,778	2,836
***Gordonia bronchialis* DSM 43234T**	75.17	74.04	**4,616**	3,282
***Gordonia polyisoprenivorans* VH2T**	74.84	73.89	77.07	**4,945**

Upper right, numbers of found orthologous genes; lower left, average nucleotide identities of orthologous genes. Bold numbers indicate the numbers of genes for each genome.
