# Supplementary material for: Draft genome of the Arabidopsis thaliana phyllosphere bacterium, Williamsia sp. ARP1
Source: Stand Genomic Sci. 2016 Jan 16;11:8. doi: 10.1186/s40793-015-0122-x (PMC4715301; doi:10.1186/s40793-015-0122-x)
Supplement: Additional file 3: — Average nucleotide identities between Williamsia sp. ARP1 and nearest actinomycete genomes. (PDF 54 kb) [file 40793_2015_122_MOESM3_ESM.pdf]

**Additional Table S3** - Average nucleotide identities (ANI) between *Williamsia* sp. ARP1 and nearest actinomycete genomes.

| **Species** | ***Williamsia* sp. ARP1** | ***Williamsia* sp. D3** | ***Gordonia bronchialis* DSM 43234** | ***Gordonia polyisoprenivorans* VH2** |
|---|---|---|---|---|
| ***Williamsia* sp. ARP1** | - | 72.37 | 72.95 | 72.71 |
| ***Williamsia* sp. D3** | 83.97 | - | 71.60 | 71.50 |
| ***Gordonia bronchialis* DSM 43234T** | 84.34 | 84.21 | - | 84.84 |
| ***Gordonia polyisoprenivorans* VH2T** | 84.17 | 84.19 | 84.84 | - |

Upper right, ANIb score calculated with BLAST; lower left, ANIm calculated with mummer.
